# P-135. Exploring Nutritional Status, Food Security, and Perceptions of Leprosy to Better Understand Factors Associated with Leprosy in Addis Ababa, Ethiopia

**DOI:** 10.1093/ofid/ofae631.340

**Published:** 2025-01-29

**Authors:** Alex R Berman, Elleni Zeleke, Liya Getachew, Aemon Fissha, Yosef Wubshet, Yitbarek Gebre, Biruk Debebe, Hatem Mohamed, Lawrence Dela Cruz, Shimelis Nigusse, Kidist Bobosha, Jessica K Fairley

**Affiliations:** Emory University School of Medicine, Department of General Internal Medicine, Atlanta, Georgia; Armauer Hansen Research Institute, Addis Ababa, Adis Abeba, Ethiopia; Emory University Rollins School of Public Health, Atlanta, Georgia; All African Leprosy Rehabilitation and Training (ALERT) hospital, Addis Ababa, Adis Abeba, Ethiopia; All African Leprosy Rehabilitation and Training (ALERT) hospital, Addis Ababa, Adis Abeba, Ethiopia; All African Leprosy Rehabilitation and Training (ALERT) hospital, Addis Ababa, Adis Abeba, Ethiopia; All African Leprosy Rehabilitation and Training (ALERT) hospital, Addis Ababa, Adis Abeba, Ethiopia; Emory University Rollins School of Public Health, Department of Global Health, Atlanta, Georgia; Emory University Rollins School of Public Health, Department of Global Health, Atlanta, Georgia; All African Leprosy Rehabilitation and Training (ALERT) hospital, Addis Ababa, Adis Abeba, Ethiopia; Armauer Hansen Research Institute, Addis Ababa, Adis Abeba, Ethiopia; Emory University, Division of Infectious Diseases, Atlanta, Georgia

## Abstract

**Background:**

Ethiopia reports 5,000 new leprosy cases yearly, making it the 5th highest country globally. While studies have established links between food insecurity, nutrition and leprosy, many gaps in knowledge about risk and transmission have led to stagnant case rates globally. Our study seeks to better understand links between nutrition, disease knowledge, and leprosy to better inform control strategies.

**Figure:**
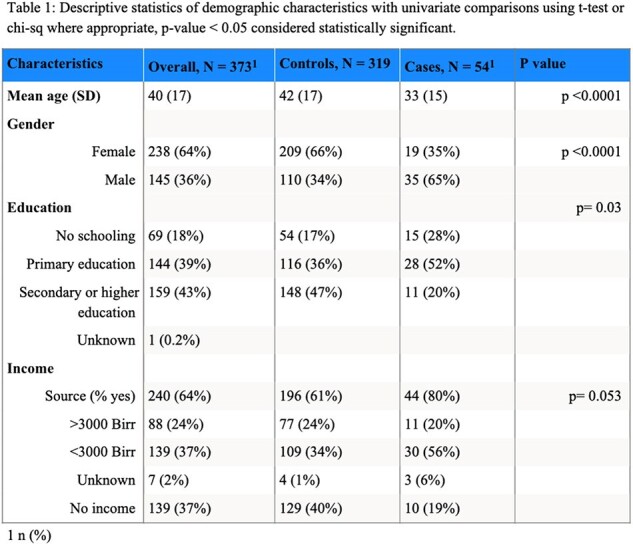

Descriptive statistics of demographic characteristics with univariate comparisons using t-test or chi-sq where appropriate, p-value < 0.05 considered statistically significant.

**Methods:**

We conducted a case-control study in adults attending the outpatient clinics of the All African Leprosy Rehabilitation and Training (ALERT) hospital. Surveys on demographics, food insecurity, and leprosy transmission knowledge were administered. Height, weight, and mid upper arm circumference (MUAC) were measured. Healthy controls were tested with a serum anti-PGL-1 antibody lateral flow assay to determine exposure to M. leprae infection. Descriptive, univariate, and multivariable logistic regression measured differences.

**Figure:**
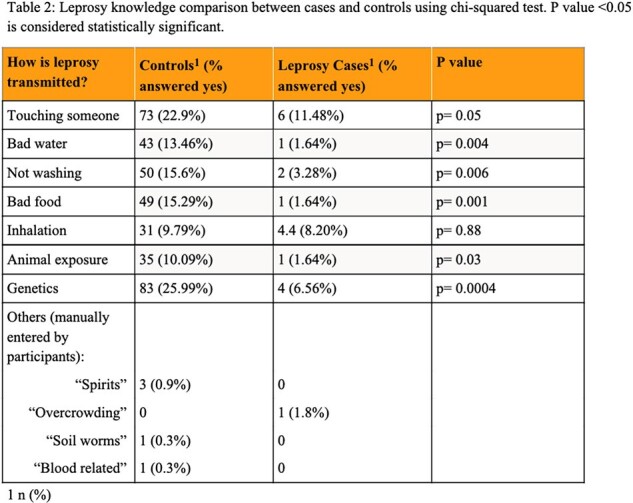

Leprosy knowledge comparison between cases and controls using chi-squared test. P value <0.05 is considered statistically significant.

**Results:**

Fifty-four individuals with leprosy and 319 controls were enrolled with more female controls than cases (66% vs 35%, p < 0.0001); and younger mean ages in cases (33 vs 42 years, p < 0.0001) (table 1). More than a third of controls were positive for anti-PGL1 antibody (n=118, 37%). The mean BMI for controls was higher than cases (23.68 vs 21.19 kg/m2, p < 0.0001), and the mean MUAC for controls was higher than cases (25.98 vs 24.37 cm, p=0.0002). Yet, cases had lower odds of being food insecure (as measured by reporting need to cut meals) when adjusted for age, sex, and SES (aOR 0.45, 95% CI 0.21, 0.98). This aOR remained similar when separating controls into anti-PGL1+ or anti-PGL1-. Cases were as likely to identify inhalation as a transmission route as controls but were less likely to identify animal exposure or hygiene practices (table 2).

**Figure:**
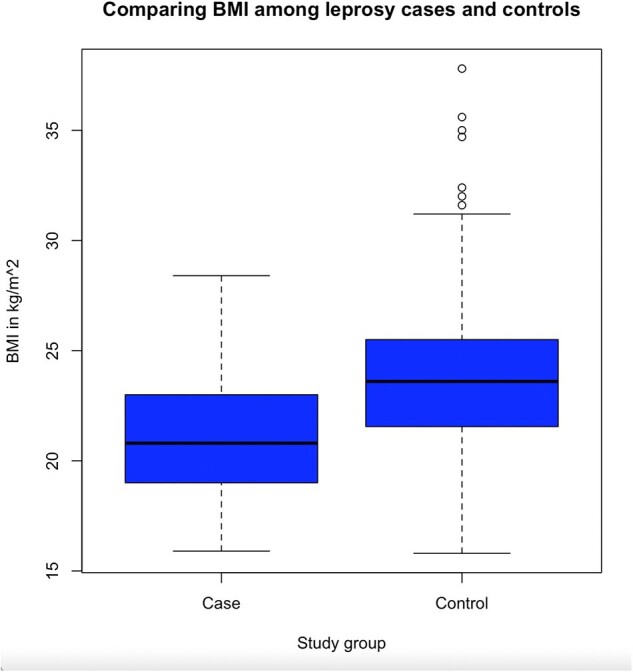

Comparing BMI among study groups using a t-test. p value <0.05 was considered statistically significant.

**Conclusion:**

Lower BMI and MUAC in cases suggest a difference in nutritional status compared to controls. But the paradoxical better food security in cases reinforces the challenges in understanding the directionality between nutritional status and disease. The knowledge results suggest a wide range of perceptions on leprosy highlighting the need for education in at-risk communities, especially given the high proportion of seropositivity in individuals living near ALERT.

**Figure:**
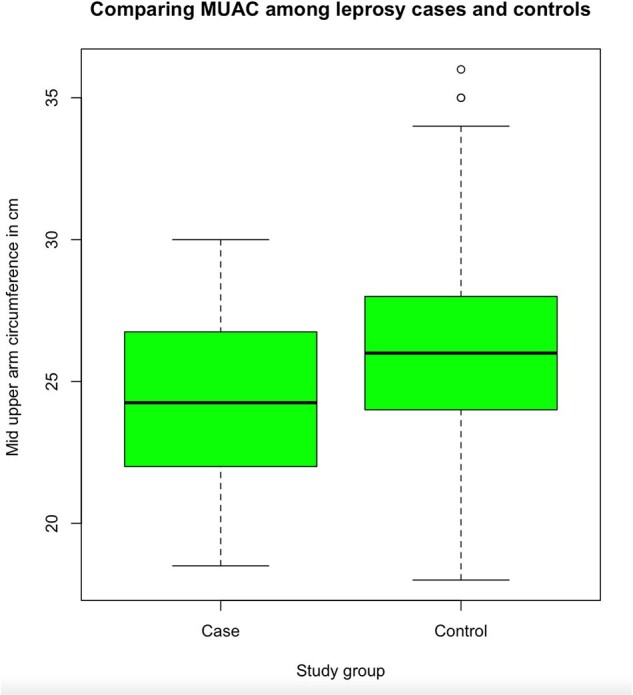

Comparing MUAC among study groups using a t-test. p value <0.05 was considered statistically significant.

**Disclosures:**

**All Authors**: No reported disclosures

